# Improving the Flexibility of Ship Propellers Additively Manufactured from High-Density Polyethylene/Long Carbon Fiber Composites by Prepreg Coating

**DOI:** 10.3390/polym16091257

**Published:** 2024-04-30

**Authors:** Gökdeniz Neşer, Ayberk Sözen, Alperen Doğru, Pengfei Liu, Erkin Altunsaray, Akile Neşe Halilbeşe, Serkan Türkmen

**Affiliations:** 1 Institute of Marine Sciences and Technology, Dokuz Eylul University, Baku Bulv. 32, Balcova, Izmir 35340, Türkiye; gokdeniz.neser@deu.edu.tr (G.N.); ayberk.sozen@deu.edu.tr (A.S.); erkin.altunsaray@deu.edu.tr (E.A.); 2 Aviation Higher Vocational School, Ege University, 1099 S. 114 Sarnic, Gaziemir, Izmir 35100, Türkiye; alperen.dogru@ege.edu.tr; 3 Marine, Offshore & Subsea Technology Group, School of Engineering, Newcastle University, Armstrong Building, Queen Victoria Road, Newcastle upon Tyne NE1 7RU, UK; pengfei.liu@ncl.ac.uk; 4 Fatsa Faculty of Marine Sciences, Ordu University, Evkaf, Arslan Aydınlık Cd. 1a, Fatsa, Ordu 52400, Türkiye; nhalilbese@odu.edu.tr

**Keywords:** composite ship propellers, HDPE/carbon fiber composites for additive manufacturing, lightweighting of ships, thermoplastic composites in marine use

## Abstract

In efforts to achieve the goal of reducing ship emissions in the fight against climate change, reducing fuel consumption by making ships lighter is stated as one of the solutions. In this study, the possibilities of making composite equivalents of propellers, which are the most complex ship elements and traditionally produced from metal materials, are investigated with the advantages of additive manufacturing, which offers a rapid production opportunity for such forms. In this way, a lighter composite propeller and, therefore, a lighter ship will be achieved, and negative environmental impacts, especially harmful emissions, will be reduced. In the study, a 1/14-scale ship propeller was produced through the material extrusion method of additive manufacturing using an HDPE composite containing long carbon fiber with a 15% weight fraction. An attempt to reduce flexibility with an epoxy-carbon fabric prepreg coating was made, as the flexibility has negative effects on the performance of the produced propeller. The propeller tunnel test showed that the applied carbon fabric epoxy prepreg helped to improve the propeller’s performance by decreasing the flexibility of the propeller and reducing the deformation at the tips. At the same time, the propeller weight was decreased by 60% compared to its metal counterparts.

## 1. Introduction

The International Maritime Organization (IMO) set a strategy to force the marine industry to reduce ship-induced greenhouse gas (GHG) emissions by at least 50% by 2050 [1]. This strategy leads to a continuous demand to improve ship performance and reduce the environmental impact during the lifecycle, which can be achieved holistically. Distinctly, this comprehensive approach addresses research areas, especially hull form optimization, novel propulsion systems applications such as energy-saving devices, new regulations, energy management systems, structural optimization, including life cycle improvements, sustainable materials, and social perceptions [2,3,4]. In these areas, it has always been desired to produce ship components with sustainable lightweight materials with high specific strength, remarkable specific stiffness, and high corrosion resistance and to make life cycle improvements.

Lightweighting, which is the only way to achieve maximum efficiency with minimum consumption, has turned into a discipline under the pressure of increasing sustainability concerns. This discipline has three basic components: lightweight materials, lightweight manufacturing, and lightweight structures.

When it comes to lightweighting materials, polymer-based composites, with their applications developing day by day since the 1950s, come to mind before their metal counterparts, such as aluminum. Carbon fiber’s performance and price, which are constantly improved by material science and engineering, attract attention. Applications that historically started with the hand lay-up method turned towards faster-capability modding processes such as injection molding, compression molding, liquid molding, and thermoforming, especially in the automotive field, in the 1980s.

Efforts to reduce the cycle time by improving the process have required the development of vacuum-assisted methods, which have also achieved improvements such as better surface quality and the elimination of in-mold coatings.

Today, resin transfer molding and structural reaction injection molding methods are used for composites in which high-performance components are used, and these processes have advantages such as reducing fiber scrap, easily coping with the form complexity of the part, even better control of the part thickness, and a relatively high processing rate.

Again, today, there is an increasing trend towards production in which hybrid multiscale composites are used (for example, using carbon nanotubes in carbon fiber composites) in terms of lightweighting [5].

However, all improvements, such as new cutting technologies, new welding technologies, additive manufacturing, and memories in general, are within the scope of lightweight manufacturing.

Additive manufacturing is also very suitable for creating lightweight structures. For example, by this method, lattice structures with varying internal density are an example of newly developed lightweight structures whose many properties are superior to those of solid materials. Additionally, with this method, topological optimization that will provide the best path for force transmission can also be easily performed [6].

Manufacturing ship components made from sustainable and lightweight polymer materials such as thermoset plastics will enable the above-mentioned improvements to be achieved. In addition to the use of lightweight materials such as aluminum and polymer-based composites, the production of complex geometries without waste is also important in this context. The prominent technology today for the manufacturing of complex geometries with the help of polymers and/or polymer composites is additive manufacturing, which has become widespread with the use of 3D printers (3DAM). Producing ship components (e.g., rudders, propellers) with sustainable and lightweight materials and 3DAM is one of the research areas that has attracted attention in recent years.

One of the main problems in the marine industry today is the unsustainability of polymer-based composites consisting of thermoset plastics, which are the most widely used materials in this industry. Because of their complex internal structure, a cost-effective end-of-life alternative has not yet been developed for them, especially in terms of recycling [7,8,9,10]. The marine industry, where thermoset composites are widely used, faces two important challenges: (1) creating end-of-life alternatives for vehicles that will not have a negative impact on the environment and (2) rapidly finding new sustainable materials and production methods due to legislation requiring the appropriate reuse or recycling of all engineering materials and products [11]. Relatively recent environmental legislation, such as EU directives for end-of-life vehicles [12] and waste electric and electronic equipment [13], requires sustainable end-of-life alternatives to thermoset plastics. By 2050, in the European Commission’s Plastics in a Circular Economy Strategy, it is stated that all plastics and composite wastes should be reused or recycled [14].

As is known, due to technological and economic difficulties, the recycling of thermoset plastics almost entirely consists of incrimination to obtain energy, resulting in no or little fiber recovery. However, although mechanical, chemical, and thermal energy conversion methods have been extensively researched for these materials, there is no widespread commercialization in this field yet.

For this reason, on a global scale, the increasing amount of unhandled end-of-life composites is directing the marine industry towards thermoplastic composites that are easily recycled by thermal methods. In the literature, there are studies on the environmental effects of composites containing new thermoplastic resins, especially on the ease of recycling and the behavior of recycled composites. For example, in a study conducted by Allagui et al. [15], it was observed that after recycling a composite produced from Elium, an innovative resin, and flax, a natural fiber, the elasticity modulus of the new composite improved while the failure properties and lifespan decreased. Additionally, in a study conducted by Sam-Daliri et al. [16], the optimization of filament and product production from glass fiber-reinforced polypropylene composite waste for material extrusion using the 3D printing method was studied.

Among the thermoplastic counterparts, high-density polyethylene (HDPE) stands out for its compatibility with marine environmental conditions, such as the following:

- Resistance to moisture and the corrosive effect of the seawater;
- Not allowing marine microorganism growth on surfaces in contact with the sea;
- High UV stability;
- Endurance under cycling loads (high fatigue strength);
- High toughness [17].

Due to the mentioned advantages, the use of HDPE in the manufacturing of many products in the marine industry, such as underwater pipes and cables, piers, small work boats, geomembranes, and cages in aquaculture farms, is becoming widespread. The most popular manufacturing methods for these products are injection molding, hot-press and material extrusion printing, and, as of recently, vacuum-assisted resin transfer molding for Elium thermoplastic resin. Since it is possible to use HDPE in 3DAM in the forms of filament, powder, and pellets, these types of production encountered are limited to some experimental studies rather than large-scale industrial applications [18,19].

One of the main reasons for the inadequacies in the use of HDPE in 3DAM is its poor printability using the material extrusion (MEX) method. In previous studies on the improvement of the printability of HDPE, it has been stated that HDPE filaments have poor adhesion to the printing surface due to their low surface energy, leading to weak interlayer bonding, warping complications during printing, and stiffness limitations compared to other engineering plastics such as PA and ABS [20]. In a study by Schirmeister et al. [19], it is noted that the printing temperature of HDPE should be kept appropriate with the help of a closed chamber, and the correct adhesive should be used in the print bed. Similarly, in a study by Jagannathan et al. [21], the printer setting, the quality of HDPE’s material properties, and the regulation of material flow were given as the key elements of HDPE’s printability and achieving a smooth product surface.

In studies carried out to improve the printability of polymer filaments, the addition of macro- and microfibers and nanoparticles comes to the forefront. In this context, glass fiber, which is affordable in cost and provides relatively moderate strength, and carbon fiber, which is relatively high in cost but provides high strength and stiffness, are preferred in new filament products, especially in industries such as the aerospace and automotive industries, where the goal is to achieve lightweight and durable structures such as car chassis, aircraft wings, frames, stringers, etc. [22,23,24,25].

Furthermore, lighter structures for the same product can be achieved thanks to the material extrusion method, which allows for thicker and more flexible blades, improving hydrodynamic performance by raising cavitation inception speeds [26]. Most sustainable material research focuses on the structural design of a plate, beam, or aircraft wing [27,28] with a mechanical performance analysis [29], failure analysis [30], optimization [31], and impact damage assessment [32]. Research on 3DAM for wing structures is focused on aircraft wings [33,34,35,36] rather than ship propellers, whose geometry is more complicated. Only Herath et al. [37] have used hydrofoil geometry in their study, but some modifications were made to the wing geometry since the geometry was not suitable for production with composite material.

In this study, a ship propeller geometry is selected as a case study that focuses specifically on sustainable materials and manufacturing systems since it is one of the fundamental components of a ship and is operated in challenging conditions such as under heavy loads and in corrosive and erosive environments. Carbon-reinforced HDPE and its coated version as sustainable polymer composites and the Fused Filament Fabrication (FFF), also known as Fused Deposition Modeling (FDM), of 3DAM as a sustainable option in the production of products with complex geometry were chosen. Experiments and numerical analyses were also performed to investigate the load-dependent deformation behavior of the blades, whose geometry was chosen to be more suitable for 3DAM [38,39].

The experimental work presented here was conducted at the Emerson Cavitation Tunnel (ECT) at Newcastle University. The results show the promising hydromechanical performance of the composite ship propeller model studied.

## 2. Materials and Methods

### 2.1. HDPE/Short Carbon Fiber Composite and Its Filament

HDPE containing 15% carbon fiber reinforcement by weight was used as the composite from which the propeller was to be produced. In fact, in studies by Hu et al. [40] and Olesik et al. [41], it is shown that both the mechanical and thermal qualities of HDPE-based composites can be improved by adding 15% reinforcement material by weight.

Extrusion and printing parameters were selected after a careful review of the relevant literature [19,20,21]. Dowaksa company’s AC4102-coded carbon fibers products (Yalova, Türkiye) whose 7 μm diameter, 6 mm fiber length; density of 1.73 g·cm^−3^ with tensile strength and modulus are 4200 MPa and 240 GPa, respectively were used as a reinforcement material. For composite production in pellet form, a 20 mm twin-screw extruder from Labtech Engineering (Samutprakarn, Thailand) was used. Arya Company’s (Izmir, Turkey) single-screw extruder, lab type, was used for filament production.

### 2.2. Prepreg Composite

A commercially available product, Kordsa’s TW245 TR30S 3K prepreg carbon epoxy fibers with an areal weight of 245 gr·m^−2^, was used to coat the surfaces of the propellers. The catalog values of the prepreg composite are given in Table 1. Prepregs were supplied in a cold chain.

### 2.3. Propeller and Its Manufacturing

**Figure 1:**
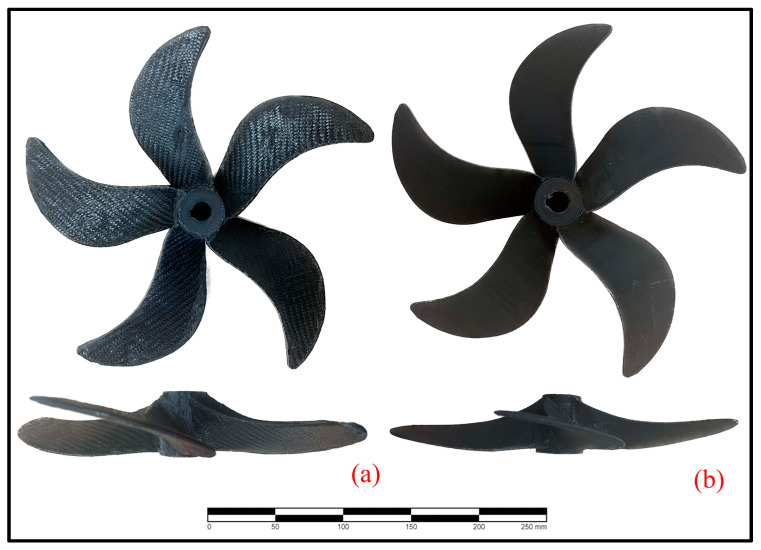
(**a**) Prepreg-coated and (**b**) uncoated propeller manufactured in the study.

The propeller, which is considered a propulsion element that is extremely suitable for AM applications with its complex form, is a 1/14-scale model of the real ship propeller, with 5 blades and a diameter of 25 cm (Figure 1). Its properties were given in Table 2.

The propeller was printed with Flashforged Creator 4 (Zhejiang, China) by using a 0.6 mm hardened steel nozzle. The print speed was 20 mm × sn^−1^, the print temperature was 230 °C, and the chamber temperature was 65 °C. The blades of the propeller were produced separately and then joined to the hub using a heat source. The propeller produced is approximately 60% lighter than its metal equivalents. In addition, the selected propeller was coated with prepreg carbon epoxy fiber with an areal weight of 300 gr × m^−2^.

### 2.4. Prepreg Coating of the Propeller

**Figure 2:**
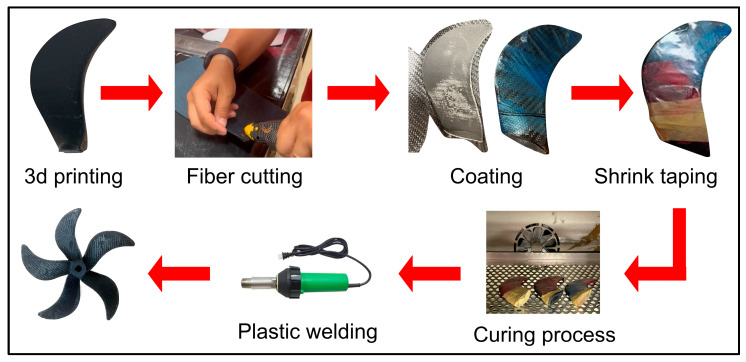
The stages of prepreg coatings of propeller blades.

Prepreg fabrics were coated on a short carbon fiber-reinforced HDPE propeller manually to reduce deflections, especially in the propeller blades’ tips, and also to make the entire propeller structure less flexible in order to improve the propeller’s performance. Prepreg pieces were cut according to the propeller geometry with fiber scissors, and then the blades were coated with the cut prepregs. Thermocouples were placed on each of the blades covered with prepreg to monitor the temperature change. All blades are wrapped with shrink tape to create a vacuum effect. When the heat was applied to the shrink tape, the tape shrank in volume and put pressure on the surface. Prepreg-coated blades were heated to 70 °C, which is the waiting temperature, at a heating rate of 1 °C × min^−1^ in an NKD240 oven of the Nükleon brand (Ankara, Türkiye). Afterwards, these blades were kept at 70 °C for 30 min, and then the temperature increased to 120 °C at a rate of 1 °C × min^−1^, and the blades were kept for 45 min. Then, after curing was completed, the part was cooled at a rate of 1 °C × min^−1^. The coating laminate was then left to cure at 80 °C. After the propeller blades were coated, the blades and the hub were heated and welded. Then, the junction corners were filled with plastic welding and sanded, as seen in Figure 2.

**Figure 3:**
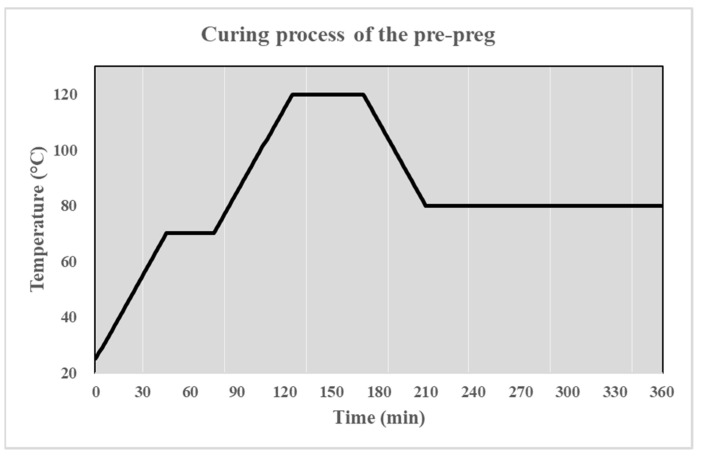
Curing process of the prepreg coating.

Temperature changes during the curing process of the prepreg coating are presented in Figure 3.

### 2.5. Mechanical Tests

**Figure 4:**
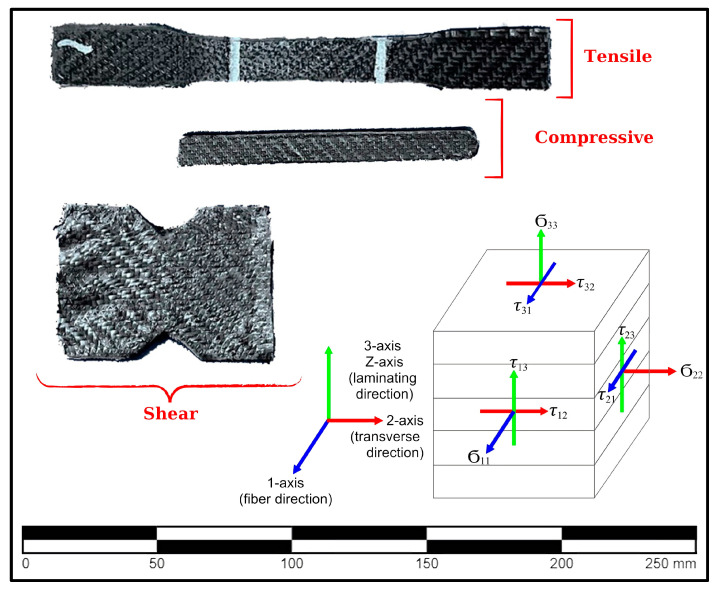
Mechanical test samples of the prepreg-composite-coated samples and the axes used.

Samples for mechanical tests were produced and prepreg-coated according to relevant standards (Figure 4). Tensile, compressive, and shear tests were performed using the Shimadzu Autograph AG-X (Tokyo, Japan), which has a 10 kN load cell and data acquisition system, at a uniform crosshead speed of 1 mm·min^−1^ in accordance with the related ASTM standards [43,44,45]. To determine the Poisson ratio and shear modulus, strain gauges from the Tokyo Measuring Instruments Lab. (Tokyo, Japan), including both one-axis and two-axis variants, were used. Data were collected using a TDS-540 data logger device (Tokyo, Japan).

### 2.6. Image Analysis

#### 2.6.1. Micro-CT Analysis

Micro-CT analysis was performed on SCANCO’s µCT model device (Brüttisellen, Switzerland). A 3 × 3 × 3 mm volume section of all specimens was scanned at 90 kVP energy, 155 µA intensity, 300 msec integration time, and 5 µm voxel size.

#### 2.6.2. Surface Electron Microscopy (SEM) Analysis

Surface electron microscopy (SEM) with a Zeiss Sigma500 FESEM (Oberkochen, Germany) and SE2 detector (Oberkochen, Germany) to inspect the surface was performed. Three-dimensionally printed specimens were cut into 20 × 20 × 30 mm rectangular prisms. Before examining the samples, they were put onto aluminum stubs with double-sided carbon tape and sputter-coated with a thin layer of 10 nm gold. The specimens were placed into the device and then exposed to a vacuum environment for four hours. They were examined at various magnifications, as indicated on the line scale, under 1.5–3.0 kV EHT, focusing on the mechanical tests’ cracks’ surface.

### 2.7. Tunnel Tests

**Figure 5:**
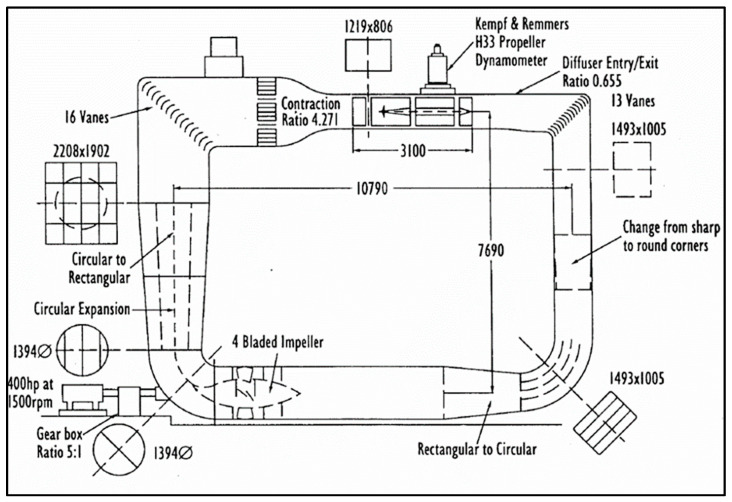
Schematic of the Emerson Cavitation Tunnel [46].

Open-water experiments were conducted at the Emerson Cavitation Tunnel (ECT) at Newcastle University (Figure 5). A main advantage of using the cavitation tunnel is that blade deflections can be observed under various loading conditions. The propeller model mounted to the tunnel’s Kempf & Remmers H45 dynamometer. Table 3 presents the main particulars of the ECT [46]. The standard ITTC procedure was followed for data acquisition [47]. During the tests, photos were taken with a Canon M50 digital DSLR camera. Images were analyzed with Rhinoceros 7 software to determine the deflection at the propeller’s tips.

Changes in the0020open-water hydrodynamic performance of the propellers were compared using the propeller advance coefficient, J.

(1) $$J=\frac{V}{n\cdot D}$$

where V is the tunnel water velocity (m·s^−1^), n is the shaft rate of the propeller (rps), and D is the propeller’s diameter. The shaft rate was kept at 16 rps, and thrust and torque data were measured at a range of advance coefficients (J) from 0.3 to 0.9. The required J values were achieved by varying the tunnel water, calculated using Equation (1).

During the tests, the tunnel parameters were arranged as in Table 4.

## 3. Results and Discussions

### 3.1. Mechanical Test Results

The results of the mechanical tests performed are given in Table 5. As can be seen from the results, the mechanical properties of the composites produced with 3DAM from HDPE material containing 15% short carbon fiber by weight could be used for a propeller. The coated composite, which achieves at least twice the improvement of the uncoated composite in terms of tensile and compressive strength and at least similar values in shear strength, represents a post-processing process that will popularize the use of 3DAM in the construction of elements with complex geometry, such as propellers.

A stiff polymer-based material will be produced in this study for the propellers, which must be traditionally produced from metallic materials whose a shape stability for reasonable performance under various loads. 

### 3.2. Results Obtained from Image Analysis

**Figure 6:**
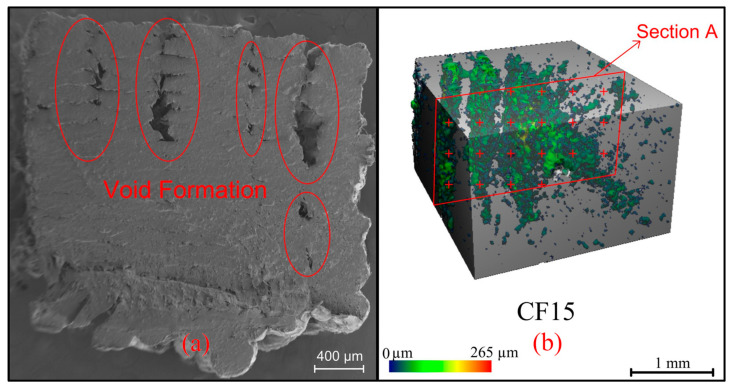
(**a**) SEM and (**b**) micro-CT images of CF15.

As shown in Figure 6, the void ratio in the sample volume is 7%, which negatively affects its mechanical properties. This result shows that the printing parameters need to be optimized. Ironing is a new pioneering method that eliminates void formation [48]. On the other hand, the SEM images show that the composite has a rough surface, which causes friction and limits the propeller’s thrust performance.

**Figure 7:**
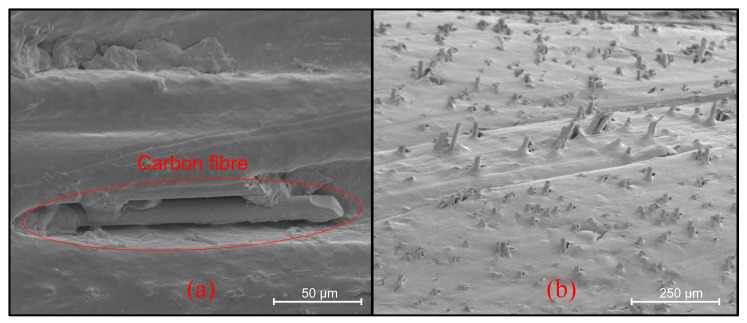
(**a**) The CF15 composite’s SEM image showing the weak bonding of HDPE and short carbon fibers as seen in red box. (**b**) Homogeneous fiber distribution on CF15 composite.

From the SEM image in Figure 7, the fibers are evenly distributed in the same direction, and the bonding of HDPE with carbon fibers is not fully realized. To eliminate this weak bonding to improve mechanical performance, it may be recommended to modify or coat with the surface of carbon fibers with some nano-additives 

### 3.3. Tunnel Test Results

**Figure 8:**
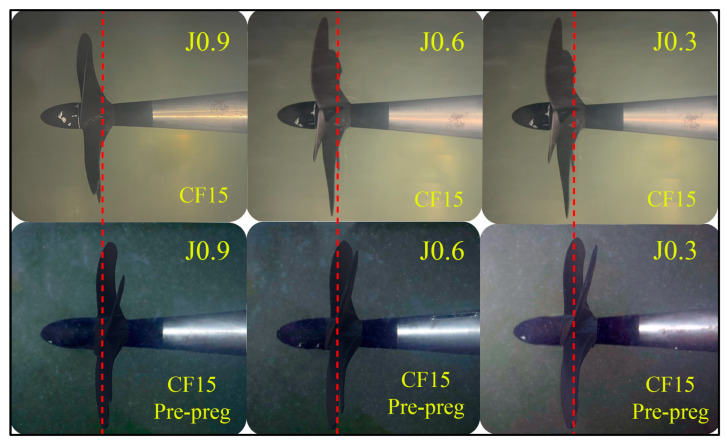
Deflections of propeller blades under various loads. Red dashed reference lines indicates first position of propeller tip.

Figure 8 and Table 6 indicate the structural responses of the propellers during the open-water tests at a range of advance coefficients (J) from 0.3 to 0.9. The vertical red line passing through the tips of the propeller blades indicates the initial position of the blades. No significant deflection occurs in the propeller at high advance ratios (J > 0.7). The deflection becomes larger under heavy load conditions (J = 0.3).

## 4. Conclusions

While HDPE-based composites are becoming more widespread day by day, improving their mechanical properties and using them in a way that brings sustainability to the forefront in every branch of industry is the subject of current research. In this study, the effect of coating one of the HDPE-based composites reinforced with short fibers with a 15% carbon content (CF15), which has shown good mechanical performance in previous studies, was studied. It is thought that coating will reduce the flexibility of final products made with HDPE-based composites, depending on the place of use. The major achievements in the study can be summarized as follows:

- The prepreg coating enabled the flexibility of the HDPE-based composite to be reduced dramatically, and thus the performance of propellers produced from this polymer composite improved by reducing the deformation at the wing tips.
- This study, which shows that it is possible to produce and improve production with the above-mentioned composite materials to lighten the propeller, which has the most complex geometry among ship elements, has the potential to produce benefits for the relevant industry.
- It has been seen that propellers, the most complex ship elements in terms of geometry, can be produced faster and more cost-effectively without the need for molding. Thus, it has been shown that the use of additive manufacturing in the marine industry can become widespread with pioneering applications such as the one in this study.
- The resulting product is also 60% lighter than its metal counterparts. This lightness will not only reduce material and labor costs and time in the production phase but will also enable the ship, which will carry a lighter propeller, to be economical throughout its operating life. The prepreg coating, a practical solution proposed in this research to improve the high level of flexibility that is a problem with composite propellers, reduced the flexibility but highlighted issues that need to be worked on, such as the propeller surface roughness. In addition, the surface improvement of carbon fibers added to HDPE material as a reinforcer stands out as an important field of study.

Future studies on the subject are suggested below:

- Interface development to improve the bonding of prepreg coatings with the surface they are coating;
- Using vacuum-assisted methods for better coating quality;
- Material and method development for surface improvement of reinforcement fibers for better bonding of components of composites to be used in additive manufacturing.

## Figures and Tables

**Table 1:** Mechanical properties of coating prepreg composite [42].

Test Performed	Property	TW245
Tensile 0°	Tensile Stress, Mpa	863
	Poisson’s Ratio	0.03
	Modulus, Gpa	58.5
Compression 0°	Compressive Stress, MPa	521.8
	Chord Modulus, Gpa	53.8
3-Point Bending	Flexural Strength, Mpa	854
	Chord Modulus, Gpa	50.9
DMA	E′ (°C)	113.2
	Tan (δ)	127.6
	E″ (°C)	124.5

**Table 2:** Main particulars of the propeller.

Propeller Type	Fixed-Pitch Propeller
Propeller diameter (D), m	0.2571
Pitch-to-diameter ratio (P/D) at 0.7R	0.83
EAR	0.466
Number of blades	5
Rake angle	0°
Skew angle (back)	14.62°
Direction of rotation	Right-handed turning
Hub-dia.-to-propeller dia. ratio	0.18
Blade thickness at 0.75R, m	0.003
Blade loading distribution (radially)	Wake-adapted
Thickness distribution	Modified after 0.8R to tip

**Table 3:** Main particulars of the Emerson Cavitation Tunnel.

Description of Facility	Vertical Plane, Closed Circulation
Test section size (L′B′H) (m)	3.10′1.26′0.80
Test section area (m^2^)	1.008
Contraction ratio	4.271
Main pump power (kW)	300
Main pump rotation speed (RPM)	294
Impeller diameter (m)	1.295
Maximum velocity (m/s)	8
Absolute pressure range (kN/m^2^)	7.6 (min)–106 (max)
Cavitation number range	0.5 (min)–23 (max)
Model propeller size (mm)	150–400

**Table 4:** Test conditions.

V (m/s)	RPM	rps	J
3.70	957.60	159.60	0.9
3.29	957.79	159.63	0.8
2.88	957.67	159.61	0.7
2.47	957.72	159.62	0.6
1.65	957.87	159.64	0.4
1.23	957.69	159.61	0.3

**Table 5:** Mechanical properties of the propellers.

Property	CF15	CF15-Prepreg
Longitudinal Young’s modulus (E_11_) (MPa)	3125	14,258
Transversal Young’s modulus (E_22_) (MPa)	3125	14,258
Longitudinal Shear modulus (G_12_) (MPa)	200	280
Transverse Shear modulus (G_13_) (MPa)	200	280
Longitudinal Poisson ratio (ν_12_)	0.44	0.26
Transverse Poisson ratio (v_23)_	0.44	0.26
Ultimate longitudinal tensile strength (Mpa)	24.42	44.47
Ultimate longitudinal compressive strength (Mpa)	18.56	30.35
Ultimate transverse tensile strength (Mpa)	24.42	44.47
Ultimate transverse compressive strength (Mpa)	18.56	30.35
Ultimate in-plane shear strength (S_12_) (Mpa)	11.56	13.67
Shear strength (S_13_) (MPa)	11.56	13.67

**Table 6:** Total deflection (mm) on propeller tips.

	CF15	CF15-Prepreg
J = 0.3	88.97	20.09
J = 0.35	82.56	18.,62
J = 0.4	76.28	17.18
J = 0.6	53.05	11.81
J = 0.7	41.35	0.91
J = 0.8	30.57	0.66
J = 0.9	17.55	0.36

## Data Availability

Data are contained within the article.
